# A Novel Cellular Model to Study Angiotensin II AT2 Receptor Function in Breast Cancer Cells

**DOI:** 10.1155/2012/745027

**Published:** 2011-12-06

**Authors:** Sylvie Rodrigues-Ferreira, Marina Morel, Rosana I. Reis, Françoise Cormier, Véronique Baud, Claudio M. Costa-Neto, Clara Nahmias

**Affiliations:** ^1^Inserm, U1016, Institut Cochin, Paris, France; ^2^CNRS, UMR 8104, Paris, France; ^3^University Paris Descartes, Paris, France; ^4^Department of Biochemistry and Immunology, Faculty of Medicine at Ribeirao Preto, University of São Paulo, 14049-900 Ribeirao Preto, SP, Brazil

## Abstract

Recent studies have highlighted the AT1 receptor as a potential therapeutic target in breast cancer, while the role of the AT2 subtype in this disease has remained largely neglected. The present study describes the generation and characterization of a new cellular model of human invasive breast cancer cells (D3H2LN-AT2) stably expressing high levels of Flag-tagged human AT2 receptor (Flag-hAT2). These cells exhibit high-affinity binding sites for AngII, and total binding can be displaced by the AT2-selective antagonist PD123319 but not by the AT1-selective antagonist losartan. Of interest, high levels of expression of luciferase and green fluorescent protein make these cells suitable for bioluminescence and fluorescence studies *in vitro* and *in vivo*. We provide here a novel tool to investigate the AT2 receptor functions in breast cancer cells, independently of AT1 receptor activation.

## 1. Introduction

Angiotensin II (AngII) is the biologically active peptide of the renin-angiotensin system, a well-known regulator of cardiovascular homeostasis and brain functions [1, 2]. Over the past ten years, a large number of studies have unraveled an additional role for the renin-angiotensin system in proliferative pathologies such as hyperplasia and cancer [3–8]. AngII binds to two major subtypes of receptors, namely, AT1 and AT2. It is generally admitted that AT1 receptor activation is responsible for most of the reported effects of AngII, whereas the AT2 receptor behaves as a negative regulator of AT1 signaling pathways [9–11]. Indeed, functional negative crosstalk between AT1 and AT2 receptors has been largely described in several physiopathological conditions including hypertension [1, 10, 11]. Activation of the AT1 receptor triggers a large number of intracellular kinases leading to modulation of cell proliferation, migration, and inflammation, three processes closely associated with tumor progression. Accordingly, several groups have shown that blocking AT1 receptors using specific receptor antagonists (ARBs) is effective in reducing tumor growth and metastasis in preclinical models [12–16]. Studies using knockout animals have further pointed out a role for stromal AT1 receptors from the host in tumor-associated macrophages infiltration and in cancer-related angiogenesis [17, 18].

A recent study [19] showing a dramatic overexpression of AT1 receptors in a subpopulation of invasive breast tumors has highlighted the potential use of ARBs as novel therapeutic agents against breast cancer. Indeed, it has been shown that losartan, an AT1 receptor blocker, was able to inhibit breast tumor growth and invasion, suggesting that effective treatments for breast cancer may be developed using drugs already used in clinics with few side effects. These exciting findings have, however, been challenged by a recent meta-analysis that suggested that ARBs medication may be associated with increased risk of cancer [20]. These provocative results have not been further validated by other groups, which rather found no effect of ARBs related to the risk of cancer [21, 22]. Nevertheless, the beneficial effects of angiotensin receptors blockade in cancer still remain a matter of debate and need to be better explored, possibly with the use of new models.

To date, most studies have mainly focused on the role of the AT1 receptor in cancer, while the role of the AT2 subtype has been largely neglected. Discrepancies in meta-analysis studies might reflect variable levels of expression of AT2 receptors in different tumors. It is important to keep in mind that antagonizing the AT1 receptor by ARBs leaves the AT2 receptor fully available for activation by local AngII. Thus, it is essential to determine whether the AT2 receptor antagonizes, or mimics, the effects of the AT1 subtype on cancer cell proliferation and invasion.

Recent studies examining the effects of AT2 receptors in cancer have remained controversial. Expression of AT2 receptors from either the tumor or stroma has been shown to attenuate the growth of pancreatic carcinoma [23], lung adenocarcinoma [24], and pheochromocytoma [25]. In contrast, other studies indicate that AT2 receptor expression associates with poor prognosis of astrocytomas [26] and that its deletion or blockade delays tumor vascularization and progression [27]. Still little is known about the effects of AT2 receptors in breast cancer. Expression of AT2 receptors in luminal epithelial cells of the normal breast has been shown to be significantly increased in breast hyperplasia and carcinomas [28]. AT2 may thus represent a new therapeutic target against breast cancer, and elucidating the functional role of these receptors in breast cancer is of major importance.

The aim of the present study was to provide a cellular model for proper investigation of the effects of the AT2 receptors in breast cancer progression.

## 2. Materials and Methods

### 2.1. Breast Cancer Cell Line

The D3H2LN cancer cell line (obtained from Caliper's) is a luciferase-expressing cell line that was derived from a spontaneous lymph node metastasis of MDA-MB-231 human breast adenocarcinoma cells [29]. D3H2LN cells were grown in DMEM 4.5 g/L glucose supplemented with 10% fetal calf serum (FCS), 1% nonessential amino acids, and 1% penicillin streptavidin and were maintained at 37°C in a 5% CO_2_ atmosphere.

### 2.2. Lentiviral Vector Construct

**Figure 1:**
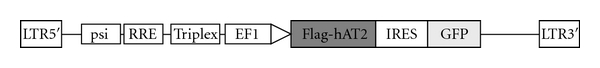
Schematic representation of the functional elements of the TRIPΔU3-EF1*α*-Flag-hAT2-IRES-GFP lentiviral vector.

The TRIPΔU3-EF1*α*-Flag-hAT2-IRES-GFP lentiviral vector (Figure 1) was constructed from a modified TRIP lentiviral vector [30, 31] by inserting the entire coding sequence of Flag-tagged human AT2 receptor (Flag-hAT2) between BamHI and XhoI restrictions sites upstream of the internal ribosomal entry site (IRES) GFP cassette. The human Flag-hAT2 sequence was amplified by PCR using the plasmid pBC-SF containing Flag-hAT2 cDNA (a generous gift of Dr. Laurent Daviet), the forward primer 5′-GCCGGATCCATGAAGACGATCATCGCCCTGAGC-3′ containing the Flag initiating codon, and the reverse primer 5′-CGCTCGAGTTAAGACACAAAGGTCTCCATTTC-3′ containing the stop codon of human AT2.

### 2.3. Lentiviral Production and Transduction

Production of infectious recombinant lentiviruses was performed by transient transfection of 293T cells as described in [31]. For infections, 3 × 10^5^ D3H2LN cells in 35 mm dishes were transduced with 5 *μ*g/mL of viral p24 (HIV-1 capsid protein). 48 h later, cells were washed, and fresh medium was added. The resulting stable cell line D3H2LN-AT2 was grown in complete medium as described above for parental D3H2LN.

### 2.4. FACS Analysis

For FACS analysis, cells were detached with PBS-EDTA 1 mM, and 10^6^ cells were fixed in paraformaldehyde 4% for 10 min. After 3 washes in PBS, GFP-positive cells were analyzed on FC-500 FACSCalibur flow cytometer using Cytomics RXP software.

### 2.5. Immunoprecipitation and Western Blotting

Cells were lysed one hour in ice with RIPA buffer containing 50 mM Tris pH7.5, 100 mM NaCl, 50 mM Na Fluoride, 0.1% SDS, 0.5% Na deoxycholate, and 1% Triton X-100 and extemporaneously supplemented by protease inhibitors (1 mM PMSF, 1 *μ*g/mL aprotinin, 5 *μ*g/mL leupeptin, and 1 *μ*g/mL pepstatin). The cell lysate was directly used for Western blotting or incubated with 2 *μ*g of anti-Flag-M2 antibody (Sigma) overnight with rotation at 4°C for Flag-AT2 immunoprecipitation. Protein G sepharose beads (50% slurry, Roche) were then added for an additional hour at 4°C. The immunocomplexes were washed three times with lysis buffer and eluted in Laemmli's sample buffer containing urea 6 M.

After one hour denaturation at 60°C, proteins and immunocomplexes were resolved on 10% SDS-PAGE and transferred to PVDF membranes. Flag-hAT2 expression was revealed using Flag-M2-HRP antibodies and enhanced chemiluminescence (ECL+, GE Healthcare).

### 2.6. Competition Binding Assays

D3H2LN-AT2 cells (3 × 10^5^ cells/well) were transferred to 12-well culture plates 24 h before binding assays. One day after plating, cells were washed briefly in 25 mM Tris-HCl buffer, pH 7.4 containing 140 mM NaCl, 5 mM MgCl_2_, and 0.1% BSA. Binding experiments were performed at 4°C to avoid any functional interference such as receptor activation, phosphorylation, or internalization. Binding was initiated by the addition of ^3^H-AngII (4pM) and increasing concentrations of nonradioactive AngII (10^−11^ M to 10^−6^ M) as competitor, in a 500 *μ*L volume of binding buffer comprising 25 mM Tris-HCl, pH 7.4, 5 mM MgCl_2_, 0.1% BSA, and 100 mg/mL bacitracin. Selective AT1 or AT2 receptor antagonists, losartan (*Galena*), and PD123319 (*Sigma*), respectively, were used at 10^−6 ^M.

## 3. Results and Discussion

The aim of this study was to generate a useful cellular model for studying the role of AT2 receptors in breast cancer. To this end, we constructed and characterized a human breast cancer cell line stably expressing high amounts of human AT2 receptors at the plasma membrane. As a recipient for AT2 receptor expression, we chose the highly aggressive D3H2LN subline derived from the well-known metastatic and triple-negative breast cancer cells MDA-MB-231 [29]. D3H2LN cells are of particular interest for the study of breast cancer cell progression both *in vitro* and *in vivo* since they are highly invasive and metastatic. After intracardiac injection into nude mice, these cells rapidly disseminate and colonize distant organs including the brain, the lungs, and the bones [29] which are the major sites of metastasis in human breast cancer. In addition, D3H2LN cells constitutively express high levels of firefly luciferase, which will be most convenient for future *in vivo* bioluminescence analysis of tumor progression and metastatic dissemination in response to AT2 receptor activation.

Preliminary experiments indicated that D3H2LN cells express very low levels of endogenous AT2 receptor transcripts as assessed by RT-PCR (data not shown) which was a prerequisite for our study. We thus designed a human AT2 receptor-containing expression vector with the objective to reach high levels of expression of the AT2 receptor and easy detection of the receptor at the cell membrane. First, to facilitate AT2 receptor detection, we used a Flag-tagged human AT2 receptor (Flag-hAT2), which can be revealed by immunofluorescence and immunoprecipitation using anti-Flag antibodies. We reasoned that by tagging the receptor at the extracellular N-terminus, we would also be able to easily detect its expression at the plasma membrane. To fulfill the other criteria and maximize the expression efficiency, the Flag-hAT2 receptor sequence was cloned into a modified TRIP lentiviral vector containing IRES-GFP (Figure 1). This lentiviral vector is of great interest since it allows high levels of AT2 receptor expression, together with concomitant expression of the green fluorescent protein (GFP) that will serve as a positive control for infection efficiency. GFP expression will also be a valuable tool for the sensitive detection of the infected cells by FACS and immunofluorescence studies.

**Figure 2:**
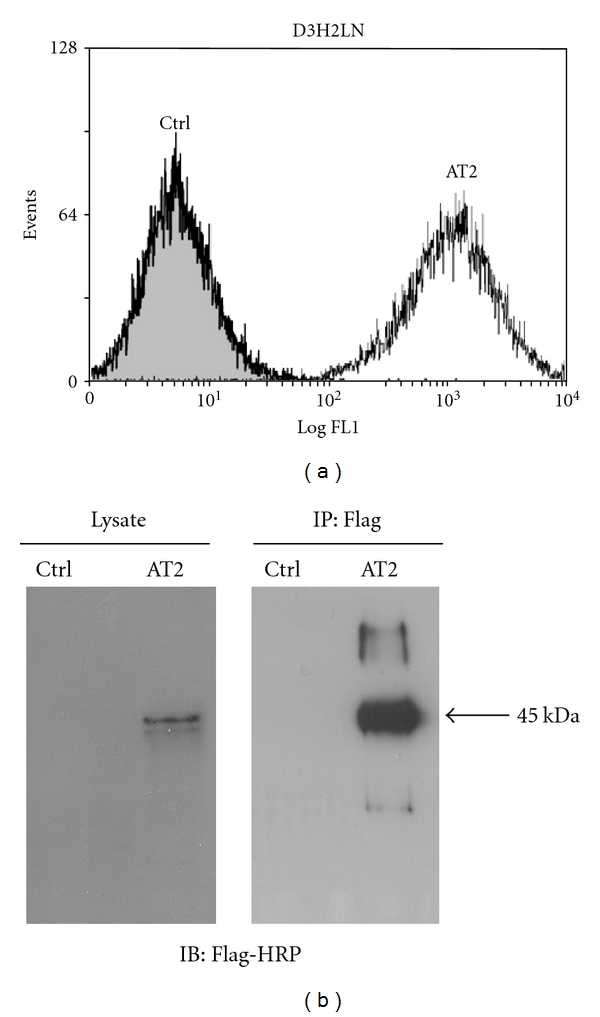
Validation of lentiviral vector transduction and expression in D3H2LN cell lines. (a) Flow cytometer analysis of GFP-positive cells. Grey-filled area represents noninfected parental D3H2LN cells (Ctrl), and white area represents infected D3H2LN-AT2 (AT2) cells. (b) Biochemical validation of Flag-AT2 expression by Western blotting (anti-Flag-HRP) in total cell lysate (left panel) or in anti-Flag immunoprecipitate fraction (right panel).

Lentiviral particles containing Flag-hAT2 were thus produced and used to transduce D3H2LN cells for 48 h. Stably infected cells maintained in culture were thereafter designated “D3H2LN-AT2” cells and further characterized. Transduction efficiency was evaluated by flow cytometry measuring GFP-positive cells. As shown in Figure 2(a), 99.5% of the cells transduced with the AT2 lentiviral vector were positive for GFP expression, indicating that virtually all infected cells had incorporated the construct. We then evaluated whether D3H2LN cells also expressed detectable amounts of the AT2 receptor. To this end, we performed Western blotting and immunoprecipitation analyses using anti-Flag antibodies. As shown in Figure 2(b) (left panel), anti-Flag-HRP antibodies revealed a major polypeptide at 45 KDa corresponding to the molecular weight of unglycosylated Flag-hAT2 receptor [32], in D3H2LN-AT2 but not in parental D3H2LN cells. Additional polypeptides of higher molecular weights (80 and 110 KDa) immunoprecipitated from D3H2LN-AT2 cells (Figure 2(b), right panel) might illustrate receptor dimerization or the presence of different glycosylated forms of the AT2 receptor [33].

 In conclusion, in the present study, we successfully isolated a stable cell line (D3H2LN-AT2) constitutively expressing the Flag-tagged human AT2 receptor and concomitantly the GFP. To note, these cells remained stable in culture after more than 15 passages (data not shown).

**Figure 3:**
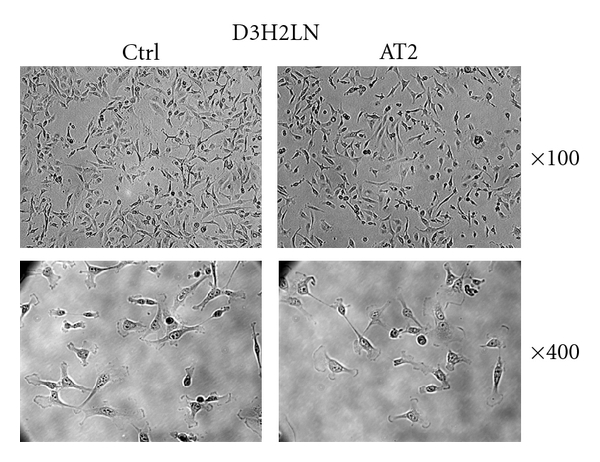
Morphological aspect of parental D3H2LN cells (Ctrl) or D3H2LN-AT2 cells (AT2). Pictures were taken under the microscope at ×100 (upper panel) and ×400 (lower panel) magnification.

To further characterize the D3H2LN-AT2 cells, culture dishes were placed under a phase contrast microscope, and pictures were taken at low (×100) and high (×400) magnification. As shown in Figure 3, there was no clear morphological differences between parental and D3H2LN-AT2 cells, indicating that overexpression of AT2 receptors in D3H2LN breast cancer cells does not significantly alter cell structure, shape, or organization.

**Figure 4:**
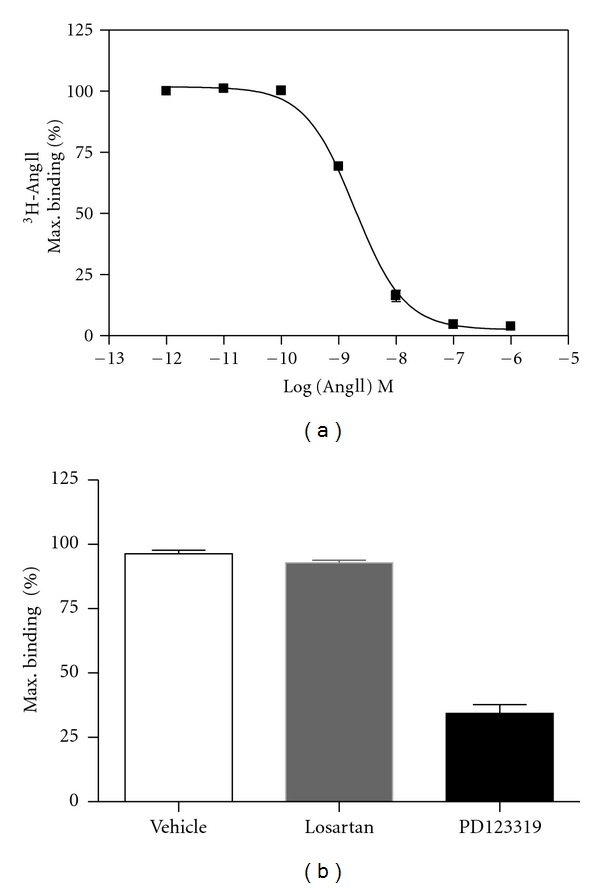
Binding studies. (a) Competition binding profile for AngII in D3H2LN-AT2 cells. Data are expressed as percentages of the maximum specific binding of the radioligand ^3^H-AngII. (b) Maximum binding obtained in the presence of the AT1 receptor antagonist (Losartan 10^−6^ M) or AT2 receptor antagonist (PD123319 10^−6^ M), as compared to the control (vehicle). Values are means ± SE of three independent experiments done in duplicate.

 We next evaluated whether the ectopically expressed Flag-hAT2 receptor was localized at the cell surface of D3H2LN-AT2 and able to bind AngII with high affinity. To address these questions, competition binding experiments were performed on intact cells with tritium labeled AngII (^3^H-AngII) in the presence of increasing concentrations of unlabelled AngII. Results revealed a classical competition binding profile in D3H2LN-AT2 cells (Figure 4(a)), indicating the presence of a single population of receptors with an IC_50_ of 1.55 ± 0.45 nM (*n* = 3) for AngII, as expected for a bona fide AngII receptor. In contrast, no specific AngII binding could be detected by binding assay in parental D3H2LN cells (data not shown), indicating that parental D3H2LN cells do not express detectable levels of AT1 and AT2 receptors in our experimental conditions.

As shown in Figure 4(b), total binding of 4pM radiolabelled AngII to D3H2LN-AT2 cells could be displaced (75%) by adding an excess (1 *μ*M) of the selective AT2 receptor antagonist PD123319 but not in the presence of an excess of the AT1 receptor antagonist losartan. These results indicate that AT2 is the major AngII binding site in D3H2LN-AT2 cells. They also suggest that overexpression of AT2 in breast cancer cells does not modulate levels of membrane AT1 receptors. Thus, ectopically expressed Flag-hAT2 receptors in D3H2LN breast cancer cells are correctly folded at the plasma membrane and are able to bind the natural agonist with the expected high affinity. Total receptor density at the cell surface was quantified to 65 pmol of receptor per 10^5^ D3H2LN-AT2 cells, which corresponds to a high level of receptors.

In conclusion, we report here the generation and characterization of a novel model of human invasive breast cancer cells (D3H2LN-AT2) that express high amounts of Flag-tagged human AT2 receptor at the plasma membrane. These cells also express GFP and luciferase, which makes them suitable for fluorescence and bioluminescence studies *in vitro* and *in vivo*. Of interest, D3H2LN-AT2 cells do not express detectable AT1 binding sites, as evaluated by radioligand binding assay, therefore allowing the characterization of AT2 functions independently of those related to AT1 receptor activation, which is of great interest in the context of AT1 blockade by ARBs.

Breast cancer is a leading cause of death by malignancy in women worldwide, and identifying new personalized therapeutic targets to fight this disease is a challenge for the coming years. Angiotensin receptors, being exposed at the plasma membrane, are easily targetable by selective receptor agonists or antagonists that may represent new and potent anticancer drugs. With the emergence of novel nonpeptidic selective agonists of the AT2 receptor such as compound 21 [34, 35], reliable tools are now available to evaluate the effects of this receptor *in vitro* and *in vivo*. There is urgent need to determine the effects of the AT2 receptor subtype in breast cancer. The cellular model presented here offers a unique opportunity to evaluate the consequences of AT2 receptor activation and blockade on breast cancer proliferation, invasion, and migration, as well as on tumor growth and metastasis formation.
